# Vitamin D and Postoperative Recovery in Elderly Ribeirinhos—Riverside Amazon Communities with Femur Fractures

**DOI:** 10.3390/clinpract15100179

**Published:** 2025-09-28

**Authors:** Elca Silvania da Silva Abreu, Caroline Oliveira dos Anjos, Zafirah Muhammad Rahman, Renata Miyabara, Ovidiu Constantin Baltatu, Luciana Aparecida Campos

**Affiliations:** 1 Center of Innovation, Technology, and Education (CITE) at Anhembi Morumbi University, Anima Institute, Sao Jose dos Campos Technology Park, São José dos Campos 12247-016, Brazilcaroline.oliveiranjos@gmail.com (C.O.d.A.);; 2 College of Medicine, Alfaisal University, Riyadh 11533, Saudi Arabia; zrahman@alfaisal.edu; 3 Office of Graduate Studies PROPPEXI, UNINOVAFAPI University Center, Afya, Teresina 64073-505, Brazil

**Keywords:** vitamin D, femur fracture, elderly, hospitalization, Ribeirinhos, postoperative recovery

## Abstract

**Background/Objectives**: Vitamin D deficiency is prevalent in elderly populations and may impact surgical recovery. Despite the equatorial location, vitamin D insufficiency affects many elderly Ribeirinhos in the Brazilian Amazon. This study investigated whether pre-existing vitamin D supplementation influences postoperative outcomes in elderly Ribeirinhos following femur fracture surgery, while examining metabolic parameters and documenting chronic disease prevalence. **Methods**: This prospective cohort study enrolled 60 elderly Ribeirinhos patients (≥65 years) admitted for femur fracture surgery at the Regional Hospital of Lower Amazon. Twenty-two patients had participated in a vitamin D supplementation program (50,000 IU monthly, 3–6 months) prior to admission; 38 had not. Primary outcomes were hospitalization duration and serum 25-hydroxyvitamin D levels. Secondary outcomes included ionized calcium, blood glucose, and comorbidity documentation. **Results**: The supplementation group was associated with significantly shorter hospitalization (14.32 ± 0.79 vs. 22.61 ± 0.88 days, *p* < 0.0001), representing 37% reduction. Vitamin D levels were markedly higher in supplemented patients (50.26 ± 2.55 vs. 21.74 ± 0.84 ng/mL, *p* < 0.0001). Ionized calcium was elevated in the supplementation group (1.29 ± 0.01 vs. 1.08 ± 0.02 mmol/L, *p* < 0.001) without hypercalcemia. Blood glucose was lower in supplemented patients (83.8 ± 1.4 vs. 152.2 ± 9.6 mg/dL, *p* < 0.001). Medical records revealed that 73.3% had hypertension and 31.7% had diabetes, with 25% having both conditions. **Conclusions**: Pre-existing vitamin D supplementation was associated with reduced hospitalization duration following femur fracture surgery, though causality cannot be inferred due to observational design. Community-based vitamin D programs may warrant further investigation in vulnerable populations, with randomized trials needed.

## 1. Introduction

Healthcare delivery in rural riverside Amazon communities, known as Ribeirinhos, presents unique challenges due to geographical isolation and limited access to medical facilities [1,2]. The Ribeirinhos, traditional riverside populations whose livelihoods depend on fishing and small-scale agriculture, face substantial barriers to accessing regular healthcare services. These communities, scattered along the Amazon river systems, often require hours or days of boat travel to reach medical facilities, resulting in delayed diagnosis and treatment of both acute and chronic conditions. Recent studies have highlighted the complex interplay between environmental, nutritional, and healthcare access factors that contribute to health disparities in these populations [3,4]. The rationale for focusing on this population stems from their unique high-risk characteristics: documented high burden of musculoskeletal disorders, paradoxical nutritional deficiencies despite tropical location, limited healthcare access, and aging demographics, making them particularly vulnerable to poor surgical outcomes [3,4].

The burden of musculoskeletal disorders (MSDs) in Ribeirinhos communities has emerged as a significant public health concern. Macedo et al. (2024) documented that over 60% of artisanal fishermen experience chronic musculoskeletal pain, particularly affecting the lower and upper back and knees, significantly impacting their quality of life and economic productivity [3]. This high prevalence of MSDs is compounded by occupational hazards, repetitive strain from fishing activities, and limited access to preventive care and rehabilitation services. The aging demographic of these communities further exacerbates musculoskeletal health challenges, particularly increasing the risk of fractures and their complications. Hip and femur fractures represent a global health crisis in elderly populations, with particularly severe implications in resource-limited settings [5]. Recent epidemiological data indicate that hip fracture incidence continues to rise worldwide, with projections suggesting a doubling of cases by 2050, particularly in developing countries [6]. Elderly patients in rural areas experience significantly worse outcomes following hip fractures, including higher mortality rates, prolonged hospitalization, and reduced functional recovery compared to their urban counterparts [7,8,9,10]. In the context of Ribeirinhos communities, these challenges are amplified by transportation difficulties, limited rehabilitation facilities, and a lack of specialized orthopedic care.

Despite the equatorial location of the Amazon region with abundant sunlight year-round, paradoxical vitamin D insufficiency affects a significant proportion of the Ribeirinhos population. Setto et al. (2022) reported vitamin D deficiency (<20 ng/mL) in 24.6–28.5% of riverside populations [11]. This finding is attributed to multiple factors, including dietary patterns low in vitamin D-rich foods, cultural practices limiting sun exposure during peak hours, occupational patterns favoring early morning and late afternoon activities, and potential genetic variations affecting vitamin D metabolism [12,13,14].

Vitamin D’s potential effects on surgical recovery are multifaceted, including roles in calcium homeostasis, immunomodulation, and muscle function [15,16]. Recent meta-analyses have reported associations between vitamin D status and hospital length of stay, with some clinical trials showing reduced hospitalization in supplemented surgical patients [17,18,19]. For example, trials have reported reductions of 3 days and 1.5 days [20,21]. However, recent large randomized controlled trials examining fracture prevention have shown different results. The VITAL [22] and DO-HEALTH [23] trials found no effect of vitamin D supplementation on fracture risk in older adults. LeBoff et Bischoff-Ferrari further concluded that these null findings may not apply to individuals with very low 25(OH)D levels [24]. These studies enrolled generally vitamin D-replete participants, highlighting the need for targeted studies in high-risk, deficient groups like the Ribeirinhos.

Despite accumulating data supporting vitamin D treatment in older surgery patients, knowledge gaps persist, especially in susceptible populations like the Ribeirinhos. Most research has been performed in well-resourced urban hospital settings, limiting its applicability to rural, resource-constrained areas [25]. Optimal dosing regimens, timing of supplementation, and population-specific considerations for tropical populations remain understudied.

Recognizing these challenges, the Regional Hospital of Lower Amazon initiated a community-based vitamin D supplementation program in January 2024, providing monthly doses of 50,000 IU cholecalciferol to elderly patients identified as high-risk for deficiency. This study aimed to investigate the association between pre-existing vitamin D supplementation and postoperative outcomes in elderly Ribeirinhos patients undergoing femur fracture surgery. We hypothesized that patients with pre-existing supplementation would show associations with improved clinical outcomes, including shorter hospitalization duration and better metabolic parameters compared to non-supplemented patients.

## 2. Methods

### 2.1. Study Design and Settings

This prospective cohort study was conducted at the Regional Hospital of Lower Amazon (HRBA, Hospital Regional do Baixo Amazonas) in Santarém, Pará, Brazil (2°26′35″ S, 54°42′30″ W), from May 2024 to November 2024. The HRBA is a public tertiary hospital under Brazil’s Unified Health System (SUS), serving as a regional referral center for advanced care to 1.4 million people across over 30 municipalities in the western Amazon [26]. The study protocol was approved by the institutional ethics committee (CAAE n. 69275023.8.0000.5492). Written informed consent was obtained from all participants or their legal representatives using standardized forms in Portuguese. For illiterate participants, consent was obtained through thumbprint in the presence of an impartial witness.

### 2.2. Study Population and Patient Selection

Elderly Ribeirinhos patients aged 65 years or older admitted to HRBA’s orthopedic ward for surgical treatment of femur fractures were systematically screened for eligibility within 24 h of admission. Ribeirinhos’ identity was standardized and verified through: (1) community health agent records documenting residence in riverside communities for ≥5 years; (2) self-identification as Ribeirinho; (3) primary occupation in fishing, agriculture, or extractivism. Recruitment occurred daily, including weekends and holidays, with a dedicated research nurse reviewing all new orthopedic admissions each morning using the hospital’s electronic admission system. Potentially eligible patients were approached between 08:00 and 12:00 h for initial screening, and family members were involved when patients had cognitive limitations.

### 2.3. Selection Criteria

Inclusion criteria comprised age 65 years or older verified by government-issued identification, acute femur fracture within 7 days from injury confirmed by radiographic imaging, fracture requiring surgical intervention (osteosynthesis or arthroplasty), confirmed Ribeirinhos community residence, ability to provide informed consent or availability of legal representative, and complete pre-admission medication history available through community health agent records, family health unit documentation, or patient/family interview.

Exclusion criteria included pathological fractures secondary to malignancy confirmed by imaging or biopsy, chronic kidney disease stage 4–5 with eGFR less than 30 mL/min/1.73 m^2^, known disorders of calcium metabolism including primary hyperparathyroidism with PTH greater than 88 pg/mL or confirmed sarcoidosis, current use of medications affecting vitamin D metabolism such as anticonvulsants (phenytoin, carbamazepine, phenobarbital) or glucocorticoids exceeding 5 mg prednisolone equivalent daily for more than 3 months, severe cognitive impairment with Mini-Mental State Examination score below 10 without available proxy, transfer from another hospital with prior surgical treatment, and life expectancy less than 6 months due to terminal illness.

### 2.4. Vitamin D Supplementation Protocol

The hospital’s vitamin D supplementation program provided cholecalciferol vitamin D monthly to elderly patients identified through community screening. The supplementation regimen consisted of cholecalciferol 50,000 IU monthly using oil-based soft gel capsules (Addera D3, Hypera Pharma, São Paulo, Brazil), stored at controlled temperature (15–30 °C) and protected from light. Distribution occurred monthly at community health posts by trained personnel, with supervised administration and documentation in patient medication cards. Adherence was monitored through patient cards and serum 25-hydroxyvitamin D levels at hospital admission. Supplementation duration ranged from 3 to 6 months before the fracture event, verified through dispensing records.

### 2.5. Outcome Measures

Primary outcomes were (1) length of hospitalization from surgery to discharge; (2) serum 25-hydroxyvitamin D levels at admission.

Secondary outcomes included (1) ionized calcium levels; (2) exploratory analysis of blood glucose levels during hospitalization; (3) documented prevalence of comorbidities.

### 2.6. Laboratory Analyses

Laboratory analyses were performed in the hospital’s certified clinical laboratory. Blood samples were collected at admission and on postoperative days 1, 3, 5, and at discharge. Serum 25-hydroxyvitamin D and blood glucose values reported represent the mean of all measurements taken during hospitalization (admission through discharge) for each patient. Serum 25-hydroxyvitamin D was measured using LC-MS/MS, ionized calcium by ion-selective electrode, and blood glucose by the hexokinase method. All methods followed standard clinical laboratory protocols with appropriate quality controls.

Vitamin D status was classified according to Endocrine Society guidelines as deficiency below 20 ng/mL, insufficiency 20–29 ng/mL, and sufficiency 30–100 ng/mL [27]. Hypercalcemia was defined as ionized calcium exceeding 2.5 mmol/L [28], and hyperglycemia as fasting glucose above 126 mg/dL or random glucose above 200 mg/dL [29].

### 2.7. Clinical Management Protocol

All patients received standardized perioperative care according to hospital protocols. Surgical procedures were performed within 48 h of admission when medically stable, with spinal anesthesia preferred using bupivacaine 0.5% at 15–20 mg. Surgical techniques were standardized by fracture type, with intertrochanteric fractures treated with a dynamic hip screw or proximal femoral nail, femoral neck fractures with hemiarthroplasty for displaced or cannulated screws for non-displaced fractures, and subtrochanteric fractures with cephalomedullary nail. Postoperative management included prophylactic antibiotics with cefazolin 2 g intravenously 30 min before incision, thromboprophylaxis with enoxaparin 40 mg subcutaneously daily adjusted for renal function, early mobilization with sitting on day 0, standing on day 1, and walking on day 2 with weight-bearing as tolerated, and standardized pain management using a multimodal approach with dipyrone 500 mg four times daily and tramadol 50 mg as needed. Physiotherapy was provided twice daily for 30 min sessions, and nutritional support included a standard hospital diet. Discharge criteria required hemodynamic stability for 48 h with blood pressure above 90/60 and heart rate 60–100, adequate pain control with oral medications achieving visual analog scale below 4, absence of surgical site complications, independent mobility with assistive devices for a minimum of 10 m, arranged follow-up at 2 weeks, and available family caregiver. Discharge decisions were made by treating physicians, unaware of study group allocation.

### 2.8. Data Collection

Data were collected using standardized forms by trained research nurses blinded to supplementation status. Variables included demographics, fracture characteristics, surgical details, comorbidities, medications, laboratory results, and clinical outcomes. Comorbidities were identified through medical record review, including documented diagnoses and current medications. Hypertension was defined as a documented diagnosis, antihypertensive use, or blood pressure 140/90 mmHg or higher on two occasions, while diabetes was defined as a documented diagnosis, antidiabetic use, or fasting glucose 126 mg/dL or higher. Nutritional status was not assessed beyond basic dietary history, and comorbidity severity was based on medical record documentation without standardized scores. Socioeconomic background was not formally measured. Missing data management allowed less than 5% missing for primary outcomes, with multiple imputation for missing baseline characteristics and sensitivity analyses comparing complete case versus imputed datasets.

### 2.9. Statistical Analysis

While complete blinding was not feasible due to the nature of the intervention, several measures were implemented to minimize bias. Laboratory personnel analyzing blood samples were blinded to participants’ group assignment. Healthcare providers assessing discharge criteria were not informed of patients’ supplementation status. Research nurses collecting outcome data used separate forms without group identification, and statisticians received coded datasets without group labels until analysis completion. No a priori sample size calculation was performed; this was a pragmatic study using all eligible patients during the study period. Post hoc power analysis with the observed effect size (Cohen’s d = 1.2) indicated 92% power for the primary outcome. Data were analyzed using GraphPad Prism version 10.4.1 (GraphPad Software, San Diego, CA, USA). Continuous variables were expressed as mean ± standard error or median (interquartile range) as appropriate. Between-group comparisons used Student’s *t*-test or Mann–Whitney U test for continuous variables and chi-square or Fisher’s exact test for categorical variables. Due to the small sample size, planned multivariable regression analysis could not be performed. Statistical significance was set at *p* < 0.05.

### 2.10. Use of AI-Assisted Technologies in Writing

AI-Assisted Writing During manuscript preparation, AI tools were used to enhance clarity and language quality. Specifically, Microsoft Copilot (version 2024.09) and QuillBot (Premium version 2024) were used for grammar checking, sentence restructuring, and improving scientific writing clarity. All AI-generated suggestions were reviewed, verified for accuracy, and edited by the authors. The AI tools were not used for data analysis, study design, data collection, or interpretation of results. The authors take full responsibility for all content and conclusions.

## 3. Results

### 3.1. Baseline Characteristics

During the study period, 78 elderly patients with femur fractures were admitted; 18 were excluded (8 with pathological fractures, 6 with severe kidney disease, 4 unable to provide consent). The final cohort comprised 60 patients: 22 in the supplementation group and 38 controls. As shown in Table 1, groups were comparable in age and sex. Table 2 shows the BMI data. Fracture types were similar between groups, with intertrochanteric fractures most common (45.5% vs. 47.4%, *p* = 0.88).

### 3.2. Primary Outcomes

**Figure 1:**
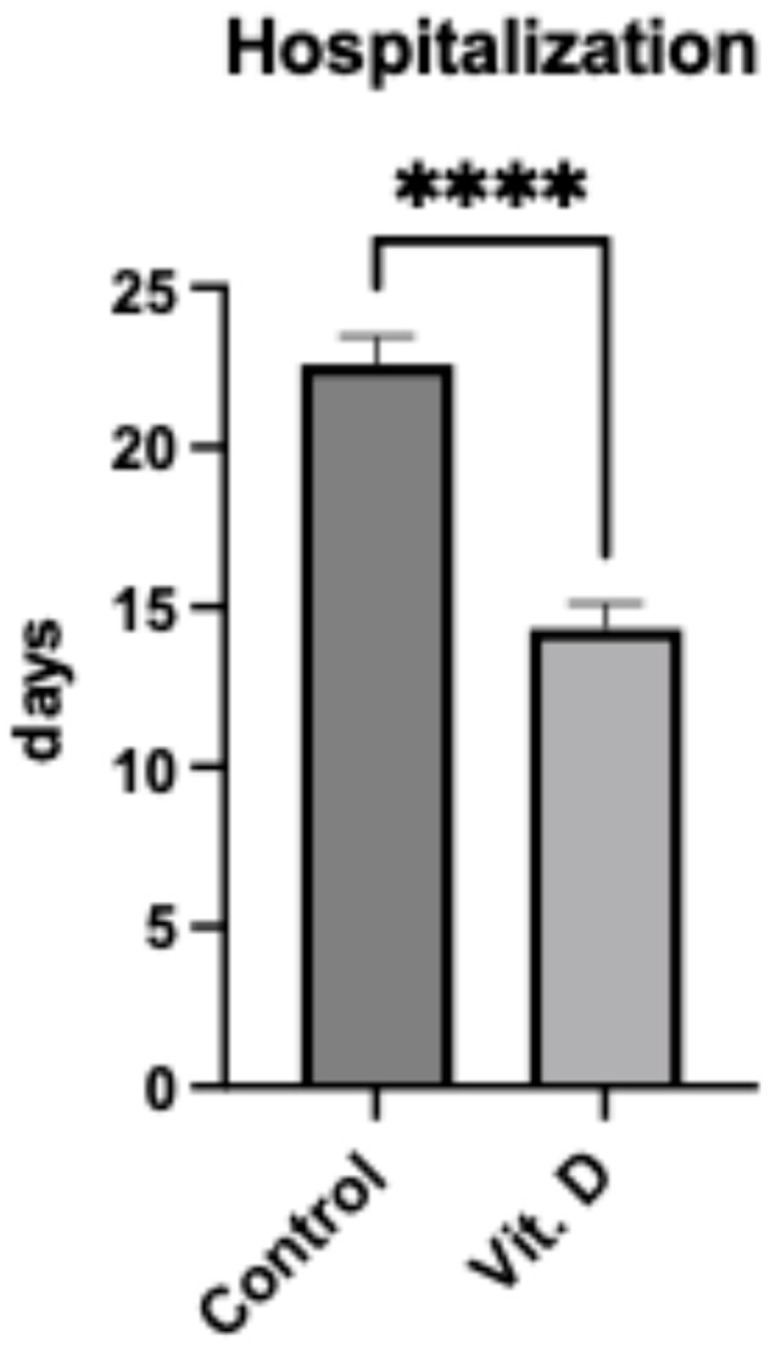
Length of hospitalization in elderly patients (*n* = 60) following femur fracture surgery. Bar graph comparing hospitalization duration (days) between control (22.61 ± 0.88 days) and Vit. D (vitamin D supplemented) (14.32 ± 0.79 days) groups. Data are presented as mean ± SEM. **** indicates *p* < 0.0001 between groups.

Length of hospitalization was significantly shorter in the supplementation group (14.32 ± 0.79 days) compared to controls (22.61 ± 0.88 days; mean difference 8.29 days, 95% CI: 5.92–10.66, *p* < 0.0001) (Figure 1). Only 2/22 (9.1%) supplemented patients required hospitalization >21 days compared to 25/38 (65.8%) controls (*p* < 0.0001).

**Figure 2:**
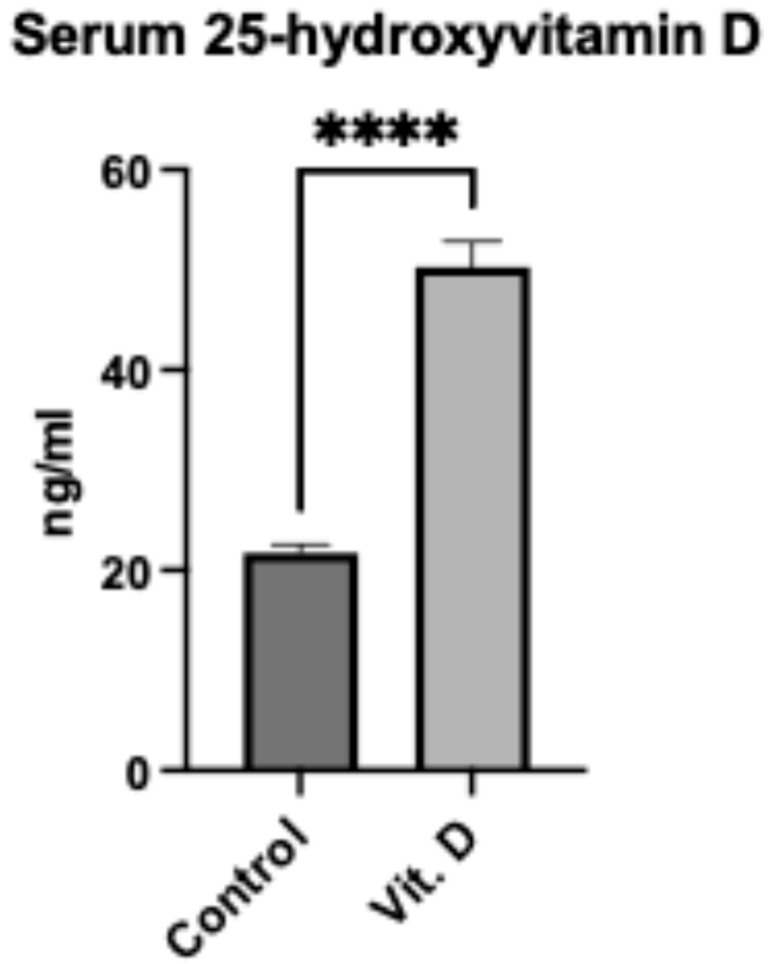
Serum 25-hydroxyvitamin D levels in elderly Ribeirinhos patients (*n* = 60) following femur fracture surgery. Bar graph comparing vitamin D concentrations between non-supplemented (21.74 ± 0.84 ng/mL) and supplemented (50.26 ± 2.55 ng/mL) groups. Data are presented as mean ± SEM. **** indicates *p* < 0.0001.

Serum 25-hydroxyvitamin D levels were significantly higher in the supplementation group (50.26 ± 2.55 ng/mL) versus controls (21.74 ± 0.84 ng/mL; mean difference 28.52 ng/mL, 95% CI: 23.17–33.87, *p* < 0.0001) (Figure 2). In the control group, 23/38 (60.5%) had vitamin D deficiency (<20 ng/mL), while all supplemented patients achieved sufficiency (≥30 ng/mL).

### 3.3. Secondary Outcomes

**Figure 3:**
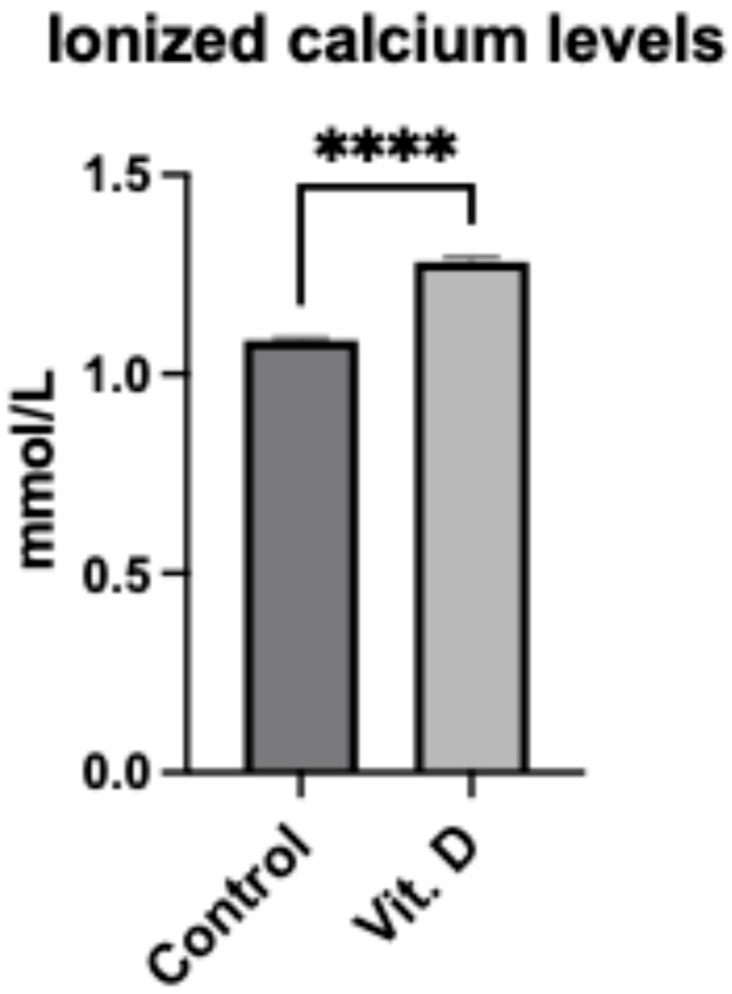
Ionized calcium levels in elderly Ribeirinhos patients (*n* = 60) following femur fracture surgery. Bar graph comparing ionized calcium levels between non-supplemented (1.08 ± 0.02 mmol/L) and supplemented (1.29 ± 0.01 mmol/L) groups. Data are presented as mean ± SEM. **** indicates *p* < 0.001.

Ionized calcium levels were higher in the supplementation group (1.29 ± 0.01 mmol/L) compared to controls (1.08 ± 0.02 mmol/L; *p* < 0.001) (Figure 3). No patients developed hypercalcemia. A positive correlation existed between vitamin D and calcium levels (r = 0.82, *p* < 0.001).

**Figure 4:**
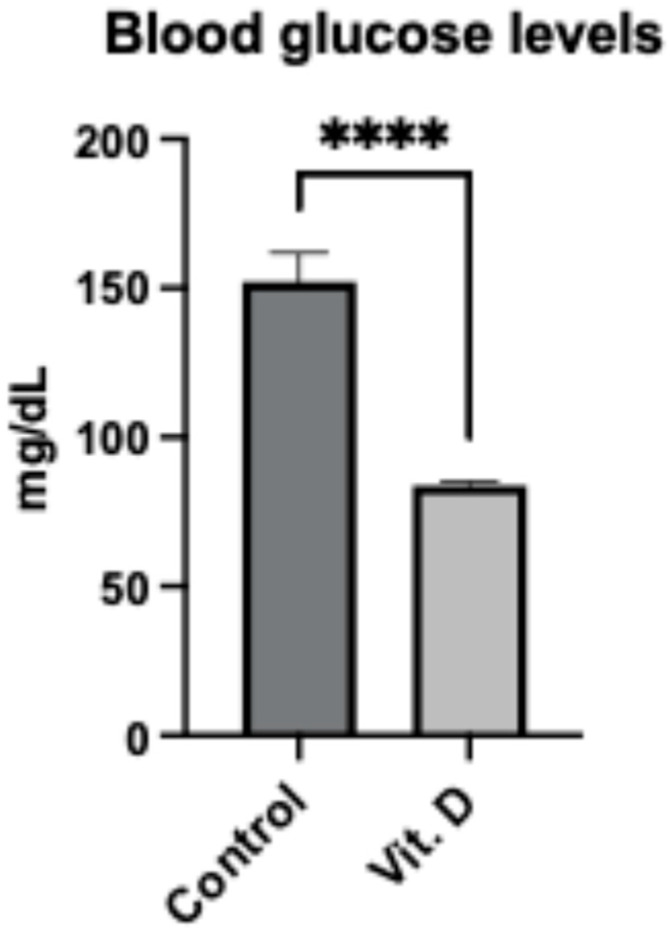
Blood glucose levels in elderly Ribeirinhos patients (*n* = 60) following femur fracture surgery. Bar graph comparing blood glucose levels between non-supplemented (152.2 ± 9.6 mg/dL) and supplemented (83.8 ± 1.4 mg/dL) groups. Data are presented as mean ± SEM. **** indicates *p* < 0.001.

Blood glucose levels during hospitalization were lower in the supplementation group (83.8 ± 1.4 mg/dL) versus controls (152.2 ± 9.6 mg/dL; *p* < 0.001) (Figure 4). However, substantial variability was noted, with a coefficient of variation of 8.2% in supplemented patients versus 24.3% in controls.

### 3.4. Comorbidity Analysis

Medical record review revealed a high prevalence of chronic conditions (Table 3). Overall, 44/60 (73.3%) had diagnosed hypertension, and 19/60 (31.7%) had diabetes. The distribution was: hypertension alone 29/60 (48.3%), diabetes alone 4/60 (6.7%), both conditions 15/60 (25.0%), neither condition 12/60 (20.0%). Between groups, hypertension prevalence was similar (supplementation: 72.7% vs. control: 73.7%; *p* = 0.94). Diabetes prevalence was also comparable (supplementation: 27.3% vs. control: 34.2%; *p* = 0.58). Chi-square analysis showed no association between hypertension and diabetes (χ^2^ = 0.126, *p* = 0.72; OR = 1.55, 95% CI: 0.41–5.89).

### 3.5. Adverse Events

No adverse events related to vitamin D supplementation were reported. Postoperative complications were similar between groups: surgical site infections (4.5% vs. 5.3%, *p* = 0.90) and thromboembolism (0% vs. 2.6%, *p* = 0.45).

## 4. Discussion

This prospective cohort study found an association between pre-existing vitamin D supplementation and reduced hospitalization duration in elderly Ribeirinhos patients following femur fracture surgery in elderly Ribeirinhos. Serum 25-hydroxyvitamin D levels were significantly higher in supplemented patients, with all supplemented individuals achieving sufficiency (≥30 ng/mL) compared to 60.5% deficiency in controls. Secondary outcomes included higher ionized calcium in supplemented patients and lower blood glucose. Comorbidity documentation revealed high rates of hypertension (73.3%) and diabetes (31.7%), with no significant between-group differences.

Our observed association aligns with previous clinical studies and meta-analyses that have indicated a link between higher vitamin D levels and better surgical outcomes, including shorter hospital stays [30,31]. However, recent large randomized controlled trials (RCTs) have not consistently demonstrated the benefits of vitamin D supplementation. The VITAL trial (*n* = 25,871) found no effect on fracture risk [22], and the DO-HEALTH trial showed no benefit for fracture prevention [23]. The discrepancy between our findings and these RCTs may reflect differences in study populations and endpoints. The major trials examined fracture prevention in generally vitamin D-replete populations, while our study assessed postoperative recovery in a population with 60.5% frank deficiency. Additionally, fracture prevention and postoperative recovery represent distinct biological processes—the former requiring long-term effects on bone density, the latter involving acute healing processes. However, these explanations remain speculative without randomized trial evidence in similar populations.

The higher ionized calcium levels in supplemented patients (1.29 vs. 1.08 mmol/L) were expected, given vitamin D’s role in calcium absorption, and remained within physiological ranges. Vitamin D’s role in calcium absorption and metabolism is well established, and optimal calcium homeostasis is essential for neuromuscular function and bone healing [32,33,34]. Vitamin D also supports muscle strength for recovery, which is critical for postoperative mobilization and rehabilitation [34,35], and may enhance immune function to reduce infection and inflammation risks [36].

The association between vitamin D supplementation and improved glycemic control aligns with evidence suggesting that vitamin D influences insulin sensitivity and pancreatic β-cell function [37,38]. These metabolic improvements may collectively contribute to enhanced tissue repair and recovery [39]. However, the observed association with lower blood glucose requires cautious interpretation. Perioperative glycemic control is influenced by numerous unmeasured factors, including baseline glycemic status, surgical stress, medications, and nutritional intake [40,41]. Without adjustment for these confounders, the glucose findings should be considered exploratory only.

### 4.1. Comorbidity Analysis

The prevalence rates of hypertension (73.3%) and diabetes (31.7%) reported here are higher than those previously observed in rural Amazon communities. Siqueira et al. [4] reported rates of 17.4% for hypertension and 6% for diabetes, while Arrifano et al. [42] found rates of 38% and 28%, respectively. This likely reflects that our elderly, hospitalized cohort represents a sicker subset of the population, and comprehensive evaluation during admission may have identified previously undiagnosed conditions.

### 4.2. Study Limitations and Strengths

Several limitations must be acknowledged. First, the observational design limits causal inference. Unmeasured confounders, including baseline functional status, nutritional markers, and socioeconomic factors, may have influenced outcomes. Second, we lacked comprehensive baseline data on renal function, albumin levels, and sarcopenia prevalence, which are factors that could affect both vitamin D metabolism and surgical recovery [43,44]. Third, the sample size, while adequately powered for primary outcomes, was insufficient for multivariable analyses or subgroup comparisons. Fourth, selection bias may exist if healthier patients were more likely to participate in the supplementation program. Fifth, we did not assess long-term outcomes, functional recovery, or quality of life measures.

The single-center design in a specific geographic and cultural context limits generalizability. Ribeirinhos populations have unique dietary patterns, sun exposure habits, and genetic backgrounds that may influence vitamin D metabolism differently than other populations [45,46,47].

Strengths include the prospective design, standardized surgical and postoperative protocols, blinded discharge decisions by treating physicians, blinded outcome assessment by research nurses and laboratory personnel, and a focus on an understudied vulnerable population.

### 4.3. Clinical Implications

While the magnitude of the observed association (8-day reduction) appears clinically meaningful, our findings should not influence current practice pending confirmation through randomized trials. The monthly 50,000 IU protocol appeared safe in achieving therapeutic vitamin D levels without hypercalcemia, but this observational evidence is insufficient to recommend routine implementation.

In resource-limited settings like rural Amazon hospitals, any intervention potentially reducing hospitalization warrants investigation [48,49]. For patients from remote communities, shorter hospitalization reduces family burden and transportation costs. However, rigorous evidence from randomized trials is essential before adopting new practices.

### 4.4. Future Directions

Our findings highlight several research needs, including randomized controlled trials in vitamin D-deficient populations to establish whether supplementation truly affects surgical outcomes, studies collecting comprehensive baseline data to enable proper adjustment for confounders, investigation of optimal dosing regimens for tropical populations, long-term follow-up to assess functional recovery and mortality, and cost-effectiveness analyses if benefits are confirmed.

### 4.5. Conclusions

This observational study found an association between pre-existing vitamin D supplementation and reduced hospitalization duration in elderly Ribeirinhos following femur fracture surgery. However, these findings must be viewed as hypothesis-generating rather than definitive. The methodological limitations, including observational design, missing baseline data, and inability to adjust for confounders, combined with contradictory evidence from large RCTs, mandate caution in interpretation. Randomized controlled trials in similar high-risk, vitamin D-deficient populations are needed before any clinical recommendations can be made.

## Figures and Tables

**Table 1:** Demographics.

Characteristic	Supplemented (*n* = 22)	Control (*n* = 38)	Total (*n* = 60)
Sex			
Female	15 (68.2%)	29 (76.3%)	44 (73.3%)
Male	7 (31.8%)	9 (23.7%)	16 (26.7%)
Age Group			
60–74 years	9 (40.9%)	14 (36.8%)	23 (38.3%)
≥75 years	13 (59.1%)	24 (63.2%)	37 (61.7%)
Ethnicity			
Brown (Pardo)	19 (86.4%)	34 (89.5%)	53 (88.3%)
Black	1 (4.5%)	3 (7.9%)	4 (6.7%)
White	2 (9.1%)	0 (0%)	2 (3.3%)
Indigenous	0 (0%)	1 (2.6%)	1 (1.7%)
Education			
Illiterate	0 (0%)	6 (15.8%)	6 (10.0%)
Elementary	10 (45.5%)	24 (63.2%)	34 (56.7%)
High School	12 (54.5%)	8 (21.0%)	20 (33.3%)

**Table 2:** Body mass index distribution.

BMI Category (kg/m^2^)	Supplemented (*n* = 22)	Control (*n* = 38)	Total (*n* = 60)
Underweight (<22.0)	4 (18.2%)	4 (10.5%)	8 (13.3%)
Normal Weight (22.0–27.0)	8 (36.4%)	11 (28.9%)	19 (31.7%)
Overweight (≥27.0)	10 (45.5%)	23 (60.5%)	33 (55.0%)

**Table 3:** Most frequent comorbidities.

Comorbidity	Supplemented (*n* = 22)	Control (*n* = 38)	Total (*n* = 60)
Hypertension	16 (72.7%)	28 (73.7%)	44 (73.3%)
Diabetes	6 (27.3%)	13 (34.2%)	19 (31.7%)
Both	5 (22.7%)	10 (26.3%)	15 (25.0%)
Neither	5 (22.7%)	7 (18.4%)	12 (20.0%)

## Data Availability

The data that support the findings of this study are available from the corresponding author upon reasonable request.
